# The Preparing Residents for International Medical Experiences (PRIME) Simulation Workshop: Equipping Surgery and Anesthesia Trainees for International Rotations

**DOI:** 10.15766/mep_2374-8265.11088

**Published:** 2021-02-11

**Authors:** J. Matthew Kynes, Rondi Kauffmann, Camila B. Walters, Christopher Sizemore, Arna Banerjee

**Affiliations:** 1 Assistant Professor, Department of Anesthesiology, Vanderbilt University Medical Center; 2 Assistant Professor, Department of Surgery, Vanderbilt University Medical Center; 3 Assistant Professor, Department of Obstetrics and Gynecology, Vanderbilt University Medical Center; 4 Associate Professor, Department of Anesthesiology, Vanderbilt University Medical Center

**Keywords:** Simulation, Global Health, Anesthesiology, Surgery, OB/GYN, Low- and Middle-Income Countries

## Abstract

**Introduction:**

Although global health training expands clinical and sociocultural expertise for graduate medical trainees and is increasingly in demand, evidence-based courses are limited. To improve self-assessed competence for clinical scenarios encountered during international rotations, we developed and assessed a simulation-based workshop called Preparing Residents for International Medical Experiences.

**Methods:**

High-fidelity simulation activities for anesthesiology, surgery, and OB/GYN trainees involved three scenarios. The first was a mass casualty in a low-resource setting requiring distribution of human and material resources. In the second, learners managed a septic operative patient and coordinated postoperative care without an ICU bed available. The final scenario had learners evaluate a non-English-speaking patient with pre-eclampsia. We paired simulation with small-group discussion to address sociobehavioral factors, stress, and teaching skills. Participants evaluated the quality of the teaching provided. In addition, we measured anesthesiology trainees' self-assessed competence before and after the workshop.

**Results:**

The workshop included 23 learners over two iterations. Fifteen trainees (65%) completed the course evaluation, 93% of whom strongly agreed that the training met the stated objectives. Thirteen out of 15 (87%) anesthesiology trainees completed the competence survey. After the training, more trainees indicated confidence in providing clinical care with indirect supervision or independently. Mean self-assessed competency scores on a scale of 1–5 increased for all areas, with a mean competency increase of 0.3 (95% CI, 0.2–0.5).

**Discussion:**

Including simulation in a pretravel workshop can improve trainees' self-assessed competence for a variety of scenarios involving clinical care in limited-resource settings.

## Educational Objectives

By the end of this activity, learners will be able to:

1. Manage scenarios encountered in low-resource settings, such as triage of critically ill patients in a nonoperative setting, crisis situations, and perioperative complications with limited diagnostic and therapeutic options.
2. Improve their knowledge of biomedical and sociobehavioral factors affecting clinical outcomes for patients experiencing poverty and endemic infectious disease.
3. Improve their skills for educating students and other health professionals.
4. Identify and reduce barriers to personal, emotional, physical, and mental health in a high-stress, unfamiliar environment.

## Introduction

In our increasingly interconnected world, global health training can play an important role in expanding clinical expertise, social and cultural awareness, understanding of health systems, appreciation for health disparities, and other educational objectives within graduate medical education (GME).^1^ Interest in global health work has dramatically increased, with a growing number of GME programs creating opportunities for trainees to participate in electives in low- and middle-income countries (LMICs). Many students applying for anesthesiology residencies have an active interest in global health, with 38% beginning residency with international experience and 48% interested in incorporating global health into their practice.^2^ Likewise, in one survey, 73% of surgical residents were willing to participate in international electives, even if this meant using vacation time or not counting cases.^3^ In response to this high interest, up to 61% of anesthesiology and 34% of surgery training programs report having an international health experience.^4,5^ Although proposals for a strategic approach to international medical education, including programs aimed at exposing trainees to sociocultural dynamics, do exist^6–8^ and negative host perceptions regarding visiting trainee attitudes and practices have been documented,^9^ there remains a lack of standardization in the global health curriculum in GME.

The Vanderbilt University Medical Center (VUMC) anesthesiology residency program first offered an international elective to its senior residents in 2010. For the elective, residents and fellows traveled to Kijabe Hospital in Kenya to teach and be supervised by VUMC and Kijabe Hospital faculty while performing and supervising anesthetics. The VUMC general surgery and OB/GYN residency programs created similar programs in 2011 and 2018, respectively, with the goals of promoting international humanitarian interests among trainees and establishing academic and research partnerships.

Interviews with participants following their rotations found that while the overall experience was rated highly, it was common for residents to experience a 1- to 2-week period of adjustment to attain a basic level of comfort and functionality within the clinical, social, and cultural environment of the international rotation. In addition, systematic reviews have established the educational and ethical benefit of structured preparatory programs for international electives.^10^ Although participants received a basic orientation to their rotation and were encouraged to prepare individually through recommended reading and informal discussion with faculty, there was no formal instructional program on international health experiences in place. In order to address this, in 2018 we developed the VUMC Preparing Residents for International Medical Experiences (PRIME) workshop. This annual, 1-day, multidisciplinary workshop focused on relevant small-group discussion and high-fidelity simulation sessions addressing clinical and ethical issues that arise in low-resource settings. The program incorporated learning objectives based on barriers encountered in low-resource environments, including decision-making based on differing health systems, advanced disease presentations, supply limitations, medication differences, cultural norms, and language differences.^11,12^ We targeted senior-level anesthesiology, OB/GYN, and surgery trainees scheduled to participate in international rotations during the coming academic year. While other programs have been developed to prepare trainees for international electives, such as the Clinical Topics in Global Health course taught at Harvard Medical School,^13^ this program was distinct in its focus on surgery and anesthesiology resident trainees and heavy incorporation of simulation activities. The international community has placed increasing attention on worldwide deficits in surgical and anesthesia care, including the findings of the Lancet Commission on Global Surgery identifying 5 billion people as being without access to safe surgical care,^14^ and the efforts of this program hope to assist trainees in alleviating these deficits.

## Methods

### Development

Development of the course stemmed from experiences of faculty and former rotation participants at the international rotation site. One faculty member held a joint appointment at Kijabe Hospital and was well versed in the environment; all the others had visited for periods of weeks to months. Course faculty created simulation content in an iterative process, with a faculty member assigned to create the basic outline, followed by review and approval by all others prior to finalization. One of the key features was to make the course interdisciplinary, which encouraged more independent activity and forced communication among trainees who were likely to be working together during their international rotation. To accommodate this, as well as to cover as much material as possible, we selected a full-day format. The multimodal teaching format allowed a variety of topics to be addressed during the day while keeping learners engaged throughout.

Simulation 1 recognized the significant role that trauma plays in health outcomes for LMICs, where 90% of the global burden of injury-related mortality occurs.^15^ Occasionally, road traffic accidents may involve crowded buses causing mass casualty and require hospitals to call for all available providers to assist. Due to the lack of prehospital emergency management systems in the country, patients may arrive unannounced, often by private vehicle. In addition, equipment and infrastructure are less reliable than in high-income settings, with evidence that electricity may be available all of the time in only 60% of facilities.^16^

Simulation 2 highlighted limitations in advanced care capacity present in many LMICs. Critical care beds are extremely scarce in these settings, with rates 100 times less than in high-income countries.^17^ At the same time, social and economic factors often mean that patients encounter delays seeking and receiving care in these settings and may present at an advanced stage of illness.^18^ By design and out of necessity, trainees in international settings often assume clinical teaching roles that require sensitivity and practice.

The final simulation illustrated the challenges of making clinical decisions without having all of the information that trainees may be accustomed to available. Many international rotations take place in settings where English is not the primary language, and translation services may not be readily available for certain dialects. While clinical staff at Kijabe Hospital are commonly fluent in English, patients are frequently less so and may only speak Swahili or languages specific to a certain tribe. Faculty also observed that due to human resource limitations, trainees in low-resource settings are frequently called to assist with duties they may not be familiar with, such as neonatal resuscitation for anesthesia residents or C-section for surgery residents.

### Course Program

The course began with an introductory session on expectations for the workshop, including simulation preparation and an overview of global surgery and anesthesia. Group meetings and debriefing took place in a meeting room adjacent to the simulation area. From the background described above, we planned three simulation scenarios: triage of multiple trauma patients in a low-resource emergency room (Appendix A), delayed presentation of intestinal obstruction (Appendix B), and pre-eclampsia in a low-resource setting (Appendix C).

In addition to simulation activities, faculty interspersed teaching sessions on medical ethics, cultural sensitivity, basic foreign language instruction, and personal risk reduction throughout the course. The curriculum also incorporated skills sessions, with the general surgery residents practicing performing cranial burr holes in a low-resource setting using a 3D-printed model of a skull, while the anesthesiology trainees discussed unfamiliar medications used in LMICs. Three core facilitators were present throughout the workshop, with additional facilitators present for prespecified sessions based on areas of expertise.

### Equipment/Environment

We utilized multiple simulation environments to recreate the clinical setting likely to be encountered on the international rotation. Common to each was the use of medication and equipment terminology and availability reflecting the local practice. This included replacing epinephrine with adrenaline and making propofol unavailable, for instance. Simulation 1 required advance preparation in order to recreate the triage environment required in an emergency room. This simulation used two SimMan (Laerdal) adult mannequins in the room initially, with a third available on a rolling stretcher to arrive after the scenario was underway. Mannequins were dressed to appear as if they had recently been in a trauma. Technicians controlled electronic vitals boards displaying heart rate, blood pressure, oxygen saturation, and respiratory rate for each mannequin by simulation software. Facilitators communicated with colleagues at each mannequin by using radios set to different frequencies. Resuscitation and intubation equipment was available at each station, with batteries removed from one of the laryngoscopes to demonstrate resource limitation. Finally, technicians identified a light switch that could dim the room lights by at least 75% when needed. The other simulations were set up as more typical examination room or operating room arrangements. For simulation 2, we created a bowel anastomosis simulator from pig intestines and placed it into a plastic box in the open abdominal cavity of the mannequin to add realism to the surgery. For the final scenario, we utilized a SimMom (Laerdal) adult mannequin with capability for simulating C-section delivery as well as a basic NeoNatalie (Laerdal) model capable of mask ventilation and chest compressions. We provided lab values and studies, when applicable, to participants when requested for simulations 2 and 3 (Appendices D and E).

### Personnel

The course as a whole made use of multiple facilitators who were exchanged throughout the day based on area of expertise. Each simulation used two to three facilitators. Facilitators were faculty familiar with simulation training as well as clinical care in a low-resource setting. In simulation 1, we assigned one facilitator each to the two initial patients in the scenario, with an additional facilitator guiding the overall progression of the session. Upper-level resident learners filled the role of physician responders in the emergency department, and all were present from the beginning of the scenario, with up to four assigned to each patient. For the other simulations, one resident learner from each specialty filled participant roles. Technical support was provided by two simulation technicians and two patient actors.

Of note, we also included didactic and small-group discussions led by faculty presenters. Faculty with specific training and expertise in risk management, ethics, and cultural humility led small-group discussions in those areas.

### Implementation

Senior residents and fellows who opted to participate in the international elective spent 4 weeks at the international site. Up to three participants rotated at a time, with as many as 15 participating over the course of a year, often with participants from different specialties participating at the same time. In order to optimize the interaction between residents, we chose a single day to invite all participants to the course. To maximize attendance, we conducted the course on a Saturday at least 4 weeks before the first set of participants was scheduled to leave for their rotation. Due to the length of the training, we made arrangements to provide breakfast and lunch for all participants. In the week preceding the session, participants received an introductory email outlining the training along with an in-depth description on the background of the international training site and, for anesthesia residents, a link to the self-assessment (Appendix F).

We recruited course facilitators among faculty with experience working and teaching in international settings. Those leading simulation sessions received an overview of the course as well as detailed case descriptions, critical actions checklist (Appendix G), and debriefing guide (Appendix H). To open the course, the course director had all participants introduce themselves, their prior experiences working in low-resource settings, and any apprehension they might have about their upcoming rotation. Importantly, the course director emphasized that parts of the course would be particularly challenging as they had been designed to bring up issues and situations many would be unfamiliar with or would never have encountered before. The director explained that the objectives of the course focused more on situational awareness, adaptability, and leadership than on clinical care and that all feedback during the course would be formative and not count toward formal evaluations.

Although the group was large, all participants were hands-on in simulation at least once as the first scenario included everyone and the others involved two or more participants. Those not participating were able to watch the scenarios by video to provide input during debriefing. Simulation sessions generally ran for 20–25 minutes, with 15–20 minutes for debriefing. Faculty directed the debriefing using discussion questions for each scenario. Group discussion was encouraged during nonsimulation activities as well, and breaks for coffee and lunch were included throughout the day. We recorded all simulation scenarios and made them available to participants through and beyond the time of their international departure, which was up to 6 months in some cases, to enable further review.

### Course Evaluation Part I: Satisfaction Survey of All Participants

Evaluation of the course took place in two parts. We created a course evaluation for all participants using a 5-point rating scale from *poor* to *excellent* based on a template already in use for simulation evaluation at the simulation center. Survey questions related to meeting course objectives, content quality, session timing, abilities of trainers, and instruction methodologies. We also obtained qualitative feedback through free-response questions on aspects of the course that should be modified or retained in future sessions. Following the completion of their rotations, residents who had participated in the course were invited to provide feedback through an anonymous survey. The survey considered the realism and preparation value of the simulation scenarios in light of the residents' subsequent international experience (Appendix I).

### Course Evaluation Part II: Self-Reported Competency Survey of Anesthesiology Residents

Because we expected anesthesiology trainees to make up the majority of participants, we developed a pre- and postprogram subjective survey for self-reported competency specific to anesthesiology. Demographic information collected included level of training and prior experience in international clinical work. The first part of the survey presented various scenarios encountered in a low-resource setting and asked for resident confidence to manage each on a 5-point scale (1 = *only as an observer,* 5 = *as an instructor of junior colleagues*). The second part asked residents to rate themselves according to ACGME milestones in nine core anesthesiology competencies adapted to practice in a low-resource setting. Using these surveys, we were able to assess three levels of Moore, Green, and Gallis' framework for planning and assessing continuing medical education activities (i.e., participation, satisfaction, and subjective competence).^19^ We used a paired-sample *t* test to compare means between responses prior to and after the workshop for each milestone. The VUMC Institutional Review Board identified the study as exempt for ethical approval.

### Debriefing

We provided facilitators with a debriefing guide for each simulation (Appendix H). Coupled with the critical actions checklist (Appendix G), we encouraged facilitators to focus on management decisions that highlighted disparities and differences existing in caring for patients in settings outside the US. Some of these included social and economic determinants of health, human and material resource limitations, cultural and language considerations, and practicing in a way that demonstrated cultural humility and respect for local systems and providers.

## Results

### Participant and Faculty Demographics

This multidisciplinary workshop included 23 senior level residents and fellows from anesthesiology (15), general surgery (six), and obstetrics (two) over 2 years. There were only minor differences in the course content between the 2 years. Facilitators involved in some part of the course included seven anesthesiologists, two general surgeons, two obstetricians, and one expert on East African culture and language. Two of the faculty facilitators had 10 or more years of primary clinical experience in Kenya.

### Course Evaluation Part I: Satisfaction Survey of All Participants

A total of 15 participants (65% response rate) completed the course evaluation, including two obstetrics, three general surgery, and 10 anesthesiology participants. Of those who completed the survey, 93% indicated that they strongly agreed the training met the stated objectives. The most positive aspects noted by learners included the simulation scenarios, ethical discussions, introduction to the local language, and discussions regarding the differences between care at their home institution and a low-resource setting. Suggested improvements included additional hands-on demonstrations and more discussion about cultural sensitivity. Following completion of their rotations, general surgery and anesthesiology residents all agreed or strongly agreed that the mass casualty and critical care capacity scenarios were realistic and prepared them for their rotations.

### Course Evaluation Part II: Self-Reported Competency Survey of Anesthesiology Residents

Thirteen out of 15 (87%) anesthesiology residents completed both parts of the self-reported competency survey. For questions presenting scenarios encountered in a low-resource setting, there was a shift in resident responses toward increasing confidence (Figure). More residents indicated confidence to participate in clinical care with indirect supervision or independently for each scenario after the training compared to before. Mean self-assessed competency levels increased for all ACGME-related anesthesiology milestones (Table), with a mean competency increase of 0.3 (95% CI, 0.2–0.5). The largest increase was for crisis management in a low-resource setting (3.4 out of 5 pre-PRIME, 3.9 out of 5 post-PRIME).

**Figure.**
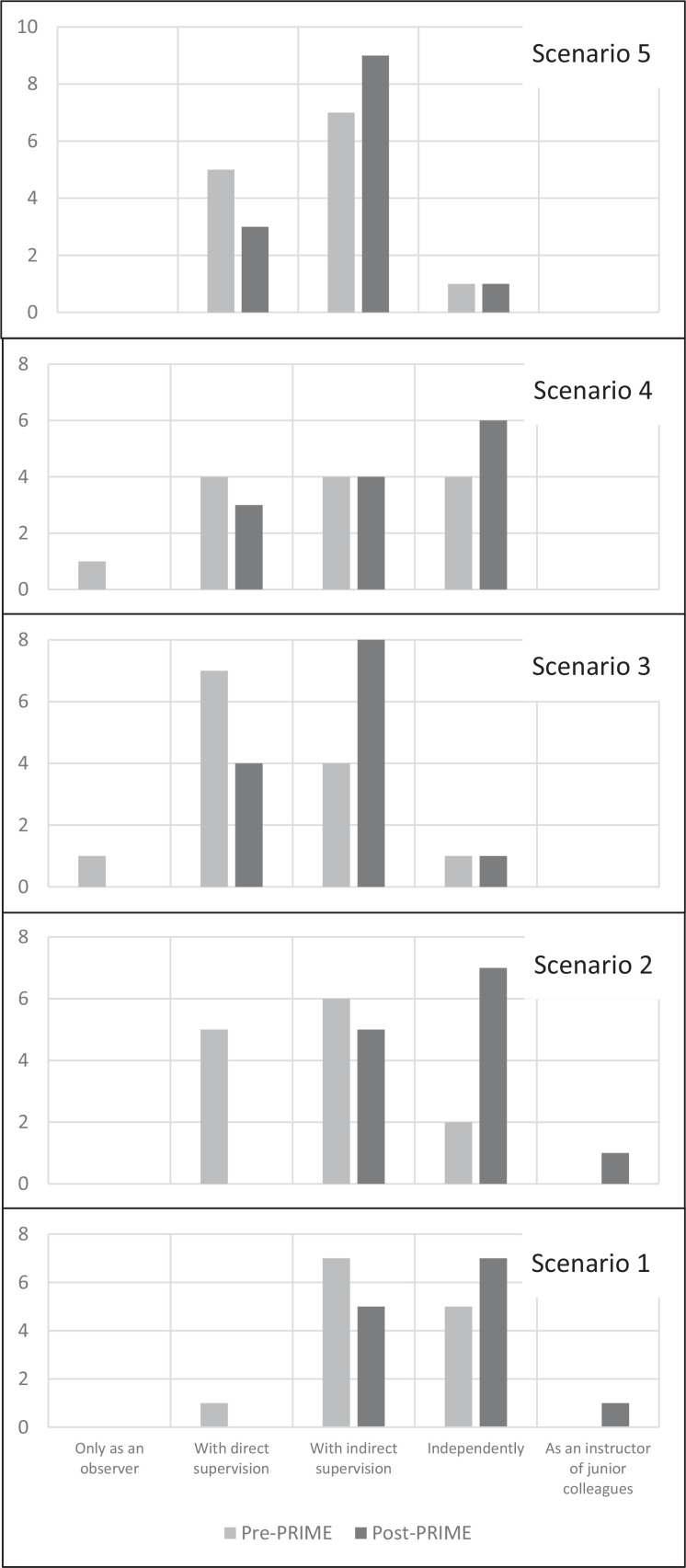
Anesthesia residents' self-reported competency level for five clinical scenarios before and after completion of the Preparing Residents for International Medical Experiences (PRIME) course. Scenario 1: exploratory laparotomy for a patient with sepsis from intestinal perforation when invasive monitoring was not available. Scenario 2: triage and stabilization of multiple trauma patients with life-threatening injuries and one available operating room. Scenario 3: general anesthesia for thoracotomy and hemorrhage control with central oxygen and power failure. Scenario 4: discussion of an accidental intrathecal injection of tranexamic acid by a junior trainee under your supervision. Scenario 5: decision-making regarding postoperative care of a postpartum patient in pulmonary edema following hemorrhage with all available ICU ventilators occupied.

**Table.**
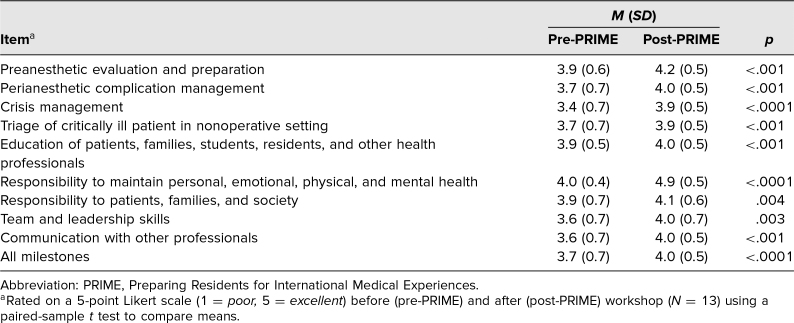
Change in Mean Resident Self-Reported Competency for ACGME Anesthesia Milestones Relevant to Providing Care in a Low-Resource Setting

## Discussion

The PRIME workshop, a novel simulation-based global surgery and anesthesiology curriculum for residents and fellows rotating in an LMIC, provided a valuable educational experience for our learners. Simulation activities in the workshop addressed issues related to health systems, advanced disease presentations, supply limitations, medication differences, cultural norms, and language differences that impacted the delivery of surgical and anesthesia care in resource-constrained settings. Evaluation forms filled out by participants in this workshop indicated a high degree of satisfaction with the training provided, as well as an increase in confidence regarding preparedness to perform at a high level during clinical encounters while abroad.

International clinical rotations present a variety of benefits to trainees, including exposure to different populations, pathogens, and pathologies often presenting at a more advanced stage than is common in high-resource settings.^8,11^ These benefits, however, come at a cost both for learners and sending institutions, which incur financial costs and safety risks associated with sending trainees abroad,^7,12,20^ and for hosting providers and institutions, which may be burdened by the impact of learners on clinical flow and decision-making.^12,21^ Improved predeparture training has been advocated by educators from both sending and hosting institutions as a means to mitigate some of these challenges,^1,22–24^ but practical descriptions of implementing such programs in surgery and anesthesiology training are scarce. The PRIME workshop aimed to help fill this gap, and its framework can be adapted to fit the needs of other programs, residents, and planned locations for global health work.

This workshop presented an opportunity to explore training strategies that reinforced a reciprocal, mutually beneficial partnership between sending and host institutions. Feedback from residents and faculty at the partner site identified specific clinical skills for our trainees to be familiar with. Facilitators emphasized that navigating differences in these environments can be very stressful, but that relying on local supervisors can ease the transition. We found that learners were enthusiastic about discussing these topics during debriefing and motivated to share the clinical and emotional challenges they encountered. Chief among the challenges referenced by participants from all specialties was the frustration they felt when required to function clinically without the information and resources they were accustomed to having in a high-resource setting. This was a common theme emphasized in all of the simulations and at times presented challenges for the facilitators, as well. Simulation 3, for example, required facilitators to move participants into potentially unfamiliar roles of assisting with C-section or neonatal resuscitation while providing enough prompting to progress through the scenario. These situations, however, provided key teaching moments for participants to consider how they might handle these frustrations in a culturally appropriate fashion when rotating at the partner site. Learners also expressed newfound appreciation for the daily challenges faced by health care providers in limited-resource settings, which may have inculcated a deeper sense of respect for their international colleagues.

Our primary outcome was participant satisfaction and self-reported confidence regarding clinical scenarios and competency. We recognized that an increase in confidence did not necessarily translate to improved clinical performance or skill level, and in the future, we plan to incorporate host institution clinical faculty evaluations of trainee skill level to assess the efficacy of our training curriculum on addressing deficiencies of our residents while rotating internationally. Additionally, pairing this workshop with other evaluation methods of participant performance, such as a faculty evaluation of scenario objective completion, in the future may be useful to identify participants in need of remediation prior to their rotation for performance below a recommended threshold.

Our curriculum was created specifically for the experiences that the trainees at our institution can anticipate during the course of their international elective in Kijabe, Kenya. As such, the curriculum may need to be modified if travel is anticipated to a different part of the world. We recognize that the local resources, customs, and culture will differ from one region of the world to another, and even from one hospital to another within the same country. One adaptation, for example, may be to include fewer monitoring resources in simulation 1 to reflect more limited equipment in certain settings and reinforce the need to directly evaluate patients through clinical exam. Our workshop faculty possessed a high level of expertise in international work, particularly at the rotation site, and used that to add depth and context to the scenarios and debriefing. However, we feel that the general objectives and content of the workshop could be met and delivered by faculty with less experience by utilizing the scenarios and debriefing guides as written.

While a strength of this course was its multidisciplinary structure, we developed surveys of self-reported competency only for anesthesiology trainees, and so, the course's effectiveness at improving confidence for surgical trainees remains unknown. In addition, although components of the self-reported competency assessments covered aspects of the educational objectives of the course, self-assessed competency cannot definitively evaluate completion of these objectives. This was a limitation, and the curriculum would be improved by better aligning objectives and evaluation methods. The sample size was also small, which may restrict the accuracy and generalizability of its findings. A larger sample size including additional training programs, long-term follow-up, and postrotation evaluation would provide a more complete picture of the efficacy of the course. Finally, while the analysis revealed statistical evidence of an increase in self-assessed competence in specific target areas, it was limited in its ability to assign a real-world educational or clinical value to what this increase means and should be interpreted accordingly.

This workshop represented a standardized curriculum for global health rotations for general surgery, obstetric, and anesthesiology residents at our institution. Participants reported high levels of satisfaction with the training, and anesthesiology participants demonstrated increased self-assessed competency and confidence in clinical care and social responsibility after the course. Given these positive findings, this course may serve as a useful model for other institutions and specialties that conduct international rotations for their trainees.

## Appendices

Simulation 1.docxSimulation 2.docxSimulation 3.docxSimulation 2 Lab Values.docxSimulation 3 Lab Values.docxResident Self-Assessment.docxCritical Actions Checklist.docxDebriefing Guide.docxSimulation Evaluation.docx
All appendices are peer reviewed as integral parts of the Original Publication.
