# Supplementary material for: The Preparing Residents for International Medical Experiences (PRIME) Simulation Workshop: Equipping Surgery and Anesthesia Trainees for International Rotations
Source: MedEdPORTAL. 2021 Feb 11;17:11088. doi: 10.15766/mep_2374-8265.11088 (PMC7880254; doi:10.15766/mep_2374-8265.11088)
Supplement: Supplementary file 1 — Simulation 1.docxSimulation 2.docxSimulation 3.docxSimulation 2 Lab Values.docxSimulation 3 Lab Values.docxResident Self-Assessment.docxCritical Actions Checklist.docxDebriefing Guide.docxSimulation Evaluation.docx [file mep_2374-8265.11088-s001.zip › A. Simulation 1.docx]

| **Appendix A: MedEdPORTAL Simulation Case 1**  **SIMULATION CASE TITLE: Triage for Multiple Trauma in a Low-Resource Emergency Room**  **AUTHORS: J. Matthew Kynes, MD, Rondi Kauffmann, MD, Arna Banerjee, MD**  **LEARNER AUDIENCE: Residents in general surgery, obstetrics/gynecology and anesthesiology** | |
| --- | --- |
| **PATIENT NAMES: Patient 1 – Unknown; Patient 2 – Alice Njeru; Patient 3 – Martin Wairegi**  **PATIENT AGE: Patient 1 – 30 years old; Patient 2 – 40 years old; Patient 3 – 50 years old**  **CHIEF COMPLAINT:**   - **Patient 1 – multiple trauma following motor vehicle crash with injuries to head, chest, arms and legs** - **Patient 2 – multiple trauma following motor vehicle crash with injury to leg and chest** - **Patient 3 – facial burn and inhalation injury following motor vehicle crash**   **PHYSICAL SETTING: Patients are arriving to an emergency room at a rural mission hospital in Kenya following a collision involving a crowded minibus.** | |
|  | |
| **Brief narrative description of case** | Up to ten learners are called urgently to an emergency room setting to assess multiple injured patients. A crowded minibus has crashed, and patients are being brought to the emergency room by private vehicles. No additional information is available. Two patients are in the trauma bay, each accompanied by a nurse who is able to provide diagnostic information. Only two monitors are available to take vital signs. Participants will be informed that additional patients are expected and evaluations must be done quickly.  The objectives of the case focus on allocation of human and material resources, triage and bringing organization to chaotic scene. Because of this, management of patients will occur in parallel. Actors and facilitators may exercise flexibility with each individual patient to cause the group to allocate and reallocate resources as the scenario progresses. |
| **Primary Learning Objectives** | 1. Triage patients according to injury severity and chance of recovery. 2. Communicate effectively among providers during crisis situations. 3. Allocate limited human and material resources with consideration for clinical need, cost and utility. 4. Manage complications of the clinical environment that may occur in low-resource settings such as power outage. |
| **Critical Actions** | Overall:   - Divide into teams to assess multiple patients in parallel - Allocate team members based on patient acuity - Reallocate human resources when the clinical scenario changes with arrival of a new patient - Adapt to resource limitations by sharing equipment and monitors, when necessary - Mobilize resources and communicate to continue to provide care during an unexpected power outage   Patient 1:   - Assign roles for initial patient assessment and treatment - Perform a primary survey (A-B-C-D-E) with intervention provided, when appropriate - Identify hypotension and administer IV fluid bolus - Identify deteriorating neurologic status after head injury and secure airway - Perform a secondary survey after primary survey is complete - Check equipment prior to intubation attempt   Patient 2:   - Assign roles for initial patient assessment and treatment - Perform a primary survey (A-B-C-D-E) with intervention provided, when appropriate - Recognize progressive tachypnea and hemodynamic collapse from tension pneumothorax - Provide appropriate chest decompression for tension pneumothorax - Perform a secondary survey after primary survey is complete   Patient 3:   - Communicate about how to reallocate staff and resources to assess and treat the new arrival - Assign roles and a perform primary survey (A-B-C-D-E) - Recognize severe inhalation injury and need for early airway intervention |
| **Learner Preparation or Prework** | The learners are working in an emergency room at a mission hospital in rural Kenya. The hospital is reasonably equipped for its setting but occasionally experiences equipment and infrastructure failure. There was a notification of a collision involving a crowded minibus with patients arriving to the hospital by private vehicles. All available hospital personnel have been called to assist with triage and stabilization of the arriving patients. Two patients are already in the emergency room – one has suffered obvious head trauma and arrived confused and hypotensive, the other has an obvious femur fracture and chest injury but otherwise appears stable. Other patients are expected to arrive at any time. |

| Initial Presentation – Patient 1 | | | |
| --- | --- | --- | --- |
| **Initial vital signs** | HR 102, BP 102/68, SpO2 90%, RR 28. Mental status: moaning in pain | | |
| **Overall Setting and Appearance** | Patient on hospital stretcher in trauma bay  Multiple traumatic injuries (leg, arm, scalp), c-collar is in place. Head injury is significant.  Monitors are connected and IV fluids are running.  Patient is initially in distress with clothes tattered indicative of recent traumatic injury.  Supplies for airway intervention and drugs for resuscitation are available. Epinephrine will be labeled as ‘Adrenaline’ and succinylcholine will be labeled “Suxamethonium.”  A chest x-ray is printed and available at bedside demonstrating three broken ribs and small hemothorax. No laboratory data is available. | | |
| **Actors (e.g., standardized participants) and their roles in the room at case start** | A nurse is immediately at bedside able to perform basic tasks under instruction from the simulation participants. The nurse can provide diagnostic clues regarding the extent of the patient’s injuries, but as an emergency patient no history is known other than the motor vehicle accident. | | |
| **HPI** | Patient was a passenger in a minibus with 15 other passengers that collided with another crowded minibus on a highway 15 miles away two hours prior. A taxi driver brought the patient to the hospital and states that the patient was alert but confused but has no other information. On arrival a c-collar was placed given the patient’s head injuries, a peripheral IV was inserted, and he was connected to monitors. No other history is available. | | |
| **Past Medical/Surgical History** | **Medications** | **Allergies** | **Family History** |
| Unknown | Unknown | Unknown | Unknown |
| **Physical Examination** | | | |
| **General** | Adult male who is confused, oriented to person but not place or time, focused on pain to head and leg, clearly in acute distress | | |
| **HEENT** | Pupils equal, round and reactive. Scalp laceration present, no intraoral injuries appreciated | | |
| **Neck** | C-collar in place and secured. Range of motion restricted due to pain. | | |
| **Lungs** | Breath sounds equal bilaterally but coarse with rales. Contusions to chest bilaterally. No stridor. | | |
| **Cardiovascular** | Progressively tachycardic and hypotensive. Normal S1, S2 with no murmur. 1+ radial, femoral and dorsalis pedis pulses. | | |
| **Abdomen** | Soft, non-distended, nontender. No organomegaly. | | |
| **Neurological** | No focal neurologic deficits. GCS: eyes open to verbal command (3), confused verbal responses (4), movements localize pain (5). Cranial nerves intact and symmetric. | | |
| **Skin** | Various abrasions to scalp, arms, and leg. | | |
| **GU** | Deferred. | | |
| **Psychiatric** | Unable to assess due to patient distress and confusion. | | |

| Instructor Notes - Changes and CASE Branch Points | | |
| --- | --- | --- |
| **Intervention / Time point** | **Change in Case** | **Additional Information** |
| Team arrival to scene with division of team members to assess and stabilize patients | Participants perform primary survey. Patient alert but confused focused on injuries to head and chest. | If asked, RN will give limited history of the patient’s obvious injuries (scalp laceration, abrasions to chest, arms and leg). |
| Team members perform primary survey | Primary survey results:  Airway – open and patent, demonstrated by patient ability to speak without obstruction.  Breathing – impaired, demonstrated by SpO2 90% and tachypnea. Stable initially but will worsen if team asks for patient to be transferred for CT scan or during secondary survey.  Circulation – impaired, demonstrated by progressive tachycardia and hypotension. IV fluid bolus should be administered and blood prepared.  Disability – impaired. Progressive confusion and patient eventually becomes obtunded until intubated.  Exposure – during primary survey team identifies injuries to head, chest, arms and leg. Patient warmed when interventions completed and progressing to secondary survey. | RN may respond, if asked, about patient injuries to head, chest and extremities.  A chest x-ray may be provided showing three broken ribs and small hemothorax. |
| Team identifies compromised air exchange during primary survey and decides to intubate | RN asks, “What drugs would you like for me to give?”  If team chooses ketamine and suxamethonium, patient will remain hemodynamically stable.  If team chooses propofol then patient will become rapidly hypotensive (60/40). If no vasopressors are administered within 2 minutes then patient will go into PEA. | ACLS should be initiated if patient progresses to PEA. Resuscitation drugs may be administered, and RN may assist with chest compressions if asked. |
| Team member begins to intubate patient. | Laryngoscope at patient bedside is not functional (battery has been removed). When this is recognized, RN may go to the other patient area to obtain a working laryngoscope.  After successful intubation, SpO2 increases to 98%. |  |
| Team provides IV fluid bolus for tachycardia during initial assessment | If fluid resuscitation given, BP and HR will remain stable.  If no fluid administered, tachycardia will progress from 102 to 110, to 130 over 5 minutes with BP decrease from 102/68 to 95/62 to 88/62 to 60/40. |  |
| Team requests ultrasound for FAST exam | RN states, “The ultrasound machine is broken.” |  |
| Team requests urgent head CT | If patient has not been intubated, RN states, “I can prepare the patient to transfer to CT.” At the same time, the SpO2 begins to decrease from 90% to 80% over two minutes with increasing RR to 34 bpm. Patient becomes more lethargic.  If patient has been intubated, RN states, “I will go inform the CT scan technician” and leaves the room. Team progresses with secondary survey. |  |
| Team requests lab studies (CBC, BMP, coagulation studies, T&S) | RN states, “I collect and send these to the lab.” |  |
| After assessing and stabilizing the patient, and while team is waiting for study results, the third patient is brought into the emergency room. | Team may choose to redistribute members to evaluate and treat newly arriving patient. RN will stay with remaining team members to assist. |  |
| After Patient 3 is assessed and airway intervention begins, there is a power outage and at least 75% of lights in the room are turned off. | Team members should identify alternative light sources (flashlights, phones, etc.) and use to guide treatment of patients. | Team must communicate about altering role responsibilities during power outage |
| Lights remain off for 2 minutes and teams continue to keep patients stabilized | If ACLS is underway, this should continue.  If patient has been intubated, they should continue to receive airway support. |  |

| Initial Presentation – Patient 2 | | | |
| --- | --- | --- | --- |
| **Initial vital signs** | HR 92, BP 112/68, SpO2 94%, RR 22. Mental status: awake, alert, complaining of pain in leg and chest | | |
| **Overall Setting and Appearance** | Patient is on hospital stretcher in trauma bay with visible injury to right leg and chest.  Monitors are connected and IV fluids are running.  Patient is initially uncomfortable but awake and alert.  Supplies for airway intervention and drugs for resuscitation are available. Epinephrine will be labeled as ‘Adrenaline’ and succinylcholine will be labeled “Suxamethonium.”  A chest x-ray is printed and available at bedside demonstrating three broken ribs and on the right side and small pneumothorax. No laboratory data is available. A lower extremity x-ray shows an isolated femur fracture. | | |
| **Actors (e.g., standardized participants) and their roles in the room at case start** | A nurse is immediately at bedside able to perform basic tasks under instruction from the simulation participants. The nurse can provide diagnostic clues regarding the extent of the patient’s injuries, but as an emergency patient no history is known other than the motor vehicle accident. | | |
| **HPI** | Patient was a passenger in a minibus with 15 other passengers that collided with another crowded minibus on a highway 15 miles away two hours prior. A taxi driver brought the patient to the hospital and states that the patient has been alert. On arrival a peripheral IV was inserted, and she was connected to monitors. Patient may volunteer that she takes no medications at home, has no chronic health conditions, and currently has pain in her chest and right leg. | | |
| **Past Medical/Surgical History** | **Medications** | **Allergies** | **Family History** |
| Prior c-section | None | None | Not contributory |
| **Physical Examination** | | | |
| **General** | An adult female complaining of pain in chest and leg, awake and alert, cooperative with exam except when needing to move leg due to pain | | |
| **HEENT** | No obvious injuries to face or oral cavity, no suspicion of head injury, pupils equal, round and reactive | | |
| **Neck** | No neck pain, no cervical injury, full range of motion | | |
| **Lungs** | Mildly decreased breath sounds on right side where pain is localized, no rales, no wheezes. | | |
| **Cardiovascular** | Pulses 2+ including right foot, hemodynamically stable initially. Normal S1, S2 with no murmur. | | |
| **Abdomen** | Soft, non-distended, nontender. No organomegaly. | | |
| **Neurological** | GCS 15, cranial nerve intact and symmetric. Oriented to person, place and time. Moves all extremities equally. | | |
| **Skin** | Abrasion to right leg, no rash, normal turgor. | | |
| **GU** | Deferred | | |
| **Psychiatric** | Follows commands and answers questions, moderately anxious and distressed | | |

| Instructor Notes - Changes and CASE Branch Points | | |
| --- | --- | --- |
| **Intervention / Time point** | **Change in Case** | **Additional Information** |
| Team arrival to scene with division of team members to assess and stabilize patients | Participants perform primary survey. Patient alert and able to give personal history. Complains of pain to right chest and right leg. | RN to assist with placing monitors on patient. |
| Team begins primary survey | Primary survey results:  Airway – patent, no obvious obstruction  Breathing – breath sounds decreased to right side with associated rib fractures, SpO2 low but stable  Circulation – adequate initially, pulses present, possible vascular injury to right femur  Disability – neurologically intact, no concern for significant head injury  Exposure – full survey completed followed by ensuring appropriate coverage of injuries |  |
| Team requests chest x-ray | RN will provide chest x-ray showing rib fractures and small pneumothorax on right side.  Even though the patient is stable, the team may decide to place a chest tube, and RN should step away to obtain supplies. Patient should remain stable until 8 minutes into the scenario. |  |
| Team recognizes relatively stable patient and redistributes members to assist with Patient 1 | When team members leave to assist Patient 1, this patient becomes progressively tachypneic and tachycardic with decrease in BP to 60/40 in 3 minutes.  Team must recognize evolving tension pneumothorax and perform needle decompression or chest tube placement.  If tension pneumothorax not recognized within 2 minutes of hypotension, patient will progress to cardiac arrest.  If chest tube already in place, then patient will remain stable.  If team decides to intubate due to declining respiratory status without recognizing tension pneumothorax, vital signs will not improve. If laryngoscope has been used for Patient 1, then team must ask RN to find another laryngoscope outside of the room. | Needle decompression should be performed to right chest using 16G angiocath.  Chest tube supplies may be gathered by RN if requested.  Resuscitation drugs may be administered during ACLS, and RN may assist with chest compressions if asked. |
| Team requests femur x-ray | RN will provide femur x-ray showing isolated fracture | If orthopedic surgery consultation requested, RN may state, “I will try to call. I know he was in a surgery when this happened.” |
| Team requests additional labs and studies (FAST, CBC, BMP, T&S, etc.) | RN may state, “I will get those sent to the lab. The ultrasound machine is broken so FAST is not available.” |  |
| After assessing and stabilizing the patient, and while team is waiting for study results, the third patient is brought into the emergency room. | Team may choose to redistribute members to evaluate and treat newly arriving patient. RN will stay with remaining team members to assist. |  |
| After Patient 3 has been assessed and airway intervention begins, there is a power outage and at least 75% of lights in the room are turned off. | Team members should identify alternative light sources (flashlights, phones, etc.) and use to guide treatment of patients. | Team must communicate about altering role responsibilities during power outage |
| Lights remain off for 2 minutes and teams continue to keep patients stabilized | If ACLS is underway, this should continue.  If patient has been intubated, they should continue to receive airway support. |  |

| Initial Presentation – Patient 3 | | | |
| --- | --- | --- | --- |
| **Initial vital signs** | HR 114, BP unknown, SpO2 88%, RR 26. Mental status: in distress, voice is hoarse | | |
| **Overall Setting and Appearance** | Patient is on hospital stretcher and rolled into the emergency room by an additional RN.  Multiple burn injuries to face, neck and upper extremities.  IV fluids are running and patient only has a portable pulse oximeter for vital signs.  Patient is struggling to breathe with a hoarse voice.  An additional set of airway management equipment (bag-valve mask, endotracheal tube, laryngoscope, suction) is made available when the patient arrives. | | |
| **Actors (e.g., standardized participants) and their roles in the room at case start** | A nurse accompanies the patient to the emergency room and is able to perform basic tasks under instruction from the simulation participants. The nurse can provide diagnostic clues regarding the extent of the patient’s injuries, but as an emergency patient no history is known other than the motor vehicle accident. | | |
| **HPI** | Patient was a passenger in a minibus with 15 other passengers that collided with another crowded minibus on a highway 15 miles away two hours prior. A taxi driver brought the patient to the hospital and the patient has just arrived. It is known that he was trapped in the burning bus for some time. | | |
| **Past Medical/Surgical History** | **Medications** | **Allergies** | **Family History** |
| Unknown | Unknown | Unknown | Unknown |
| **Physical Examination** | | | |
| **General** | An adult male patient who is awake but struggling to breathe, voice is hoarse, in acute distress | | |
| **HEENT** | Burns to face and neck, voice is hoarse, nostrils singed, oral cavity erythematous | | |
| **Neck** | Burns present, no c-collar in place, range of motion limited by pain from burns | | |
| **Lungs** | Coarse breath sounds, equal bilaterally, expiratory wheeze, tachypnea | | |
| **Cardiovascular** | Tachycardic with normal BP, 2+ pulses present throughout, normal S1, S2 with no murmur | | |
| **Abdomen** | Soft, non-distended, nontender, no organomegaly | | |
| **Neurological** | No focal cranial nerve deficits, GCS 14, moves all extremities equally, oriented to person, time and place | | |
| **Skin** | Second and third degree burns to face and neck, no rash | | |
| **GU** | Deferred | | |
| **Psychiatric** | Extremely anxious, responds with single word answers, appears in acute distress | | |

| Instructor Notes - Changes and CASE Branch Points | | |
| --- | --- | --- |
| **Intervention / Time point** | **Change in Case** | **Additional Information** |
| Patient is brought into scenario by stretcher 10 minutes after it has already begun. | Participants must decide who will leave the current patients to assess and treat the new patient. |  |
| Team requests full vital signs | RN states, “The portable pulse oximeter is the only one available right now.” | If requested, BP cuff may be taken from another patient for to obtain a BP reading. |
| Team performs primary survey | Primary survey results:  Airway – evidence of obstruction and impending collapse given patient’s effort and signs of inhalation injury and facial burns.  Breathing – impaired, demonstrated by low SpO2 and tachypnea. Supplemental oxygen may be requested without improvement.  Circulation – tachycardia, all pulses present and bounding, if BP obtain it is normal.  Disability – no neurologic deficits appreciated by exam or concern for head injury.  Exposure – no other injuries appreciated other than burn injuries to face and neck.  Team may opt to intubate when airway is evaluated due to impending airway collapse. If primary survey progresses without intervention, SpO2 should decrease to 80% over three minutes. | Oxygen should only be administered if requested.  If team continues past airway evaluation and does not notice further decrease in SpO2, RN may state, “I am concerned about the patient’s breathing. This burn to the patient’s face seems very serious.” |
| Team prepares to intubate patient due to airway compromise | Induction may proceed with ketamine, etomidate or propofol and suxamethonium or rocuronium.  Direct laryngoscopy is the only airway device available. A bougie may be provided if requested. | Induction drugs and airway supplies are available from RN. |
| Once team is prepared to induce and intubate, there is a power outage and at least 75% of lights in the room are turned off. | Team members should identify alternative light sources (flashlights, phones, etc.) and use to guide treatment of patients. | Team must communicate about altering role responsibilities during power outage |
| Lights remain off for 2 minutes and teams continue to keep patients stabilized | If patient has been intubated, they should continue to receive airway support. |  |

**Ideal Scenario Flow**

The learners will have limited introductory information other than that there has been a mass casualty from a motor vehicle crash involving a bus and that multiple patients are arriving to the emergency department. All available physicians have been called to assist. If fewer than five learners are available, then the scenario may proceed without Patient 2. On arrival, learners will divide themselves between patients to begin primary assessment and stabilization. Management of patients will proceed in parallel. If controlling multiple mannequins and monitors is technically difficult, patient progression may be guided directly by facilitators at the bedside. Patient 1 is initially the more critical patient and will require more rapid intervention with IV fluid administration for hypotension and eventually intubation for respiratory failure. Patient 2 will appear stable initially but progress to cardiopulmonary collapse due to tension pneumothorax unless a chest tube is inserted. Learners may need to share resources, such as a non-functional laryngoscope. As the learners identify the primary issue with each patient and stabilize them, a third patient arrives to the scenario. Learners will need to reallocate their resources, including monitors, to assess and stabilize this patient with rapidly progressing inhalation injury. Just as the intervention to stabilize the final patient proceeds, there is a power outage which requires learners to quickly adapt to ensure on-going safe and effective care for their patients.

**Anticipated Management Mistakes**

1. Failure to assign roles for trauma management: Some learner groups failed to identify a team leader and assign roles for assessment and intervention. Specific roles include team leader, medication administration, airway management, and others. Including the arrival of an additional patient provided an additional opportunity to assign roles and organize the team response, and this was emphasized in the debriefing.
2. Difficulty running multiple patients in parallel: Having multiple patients at the same time introduced technical and logistical challenges. Our set-up allowed for separate instructors to communicate with technicians and actors for each mannequin. However, other set-ups may require instructors to fill the role of actors and guide progression of the case at the bedside. For the third patient, we used a simulated monitor with a printed set of vital signs because we were unable to use remote access to control the screen.
3. Learner confusion during power outage: Initially, learners did not appreciate that the power outage was part of the scenario. However, keeping the majority of the lights off for two minutes forced them to adapt and find a way to provide on-going care and treatment until the issue was resolved. This was discussed in depth during the debriefing as an illustration of realities faced in low-resource settings.
