# Supplementary material for: The Preparing Residents for International Medical Experiences (PRIME) Simulation Workshop: Equipping Surgery and Anesthesia Trainees for International Rotations
Source: MedEdPORTAL. 2021 Feb 11;17:11088. doi: 10.15766/mep_2374-8265.11088 (PMC7880254; doi:10.15766/mep_2374-8265.11088)
Supplement: Supplementary file 1 — Simulation 1.docxSimulation 2.docxSimulation 3.docxSimulation 2 Lab Values.docxSimulation 3 Lab Values.docxResident Self-Assessment.docxCritical Actions Checklist.docxDebriefing Guide.docxSimulation Evaluation.docx [file mep_2374-8265.11088-s001.zip › B. Simulation 2.docx]

| **Appendix B: MedEdPORTAL Simulation Case 2**  SIMULATION CASE TITLE: Delayed presentation of intestinal obstruction  AUTHORS: J. Matthew Kynes, MD, Rondi Kauffmann, MD, Arna Banerjee, MD  LEARNER AUDIENCE: Residents in general surgery, obstetrics/gynecology and anesthesiology | |
| --- | --- |
| **PATIENT NAME:** Joseph Muchendu  **PATIENT AGE:** 10 years  **CHIEF COMPLAINT:** Fever, abdominal pain, vomiting  **PHYSICAL SETTING:** Operating room | |
|  | |
| **Brief narrative description of case** | Two learners must co-manage the surgical and anesthetic management of a child with advanced septic shock due to bowel perforation. The child’s presentation has been delayed for several days due to social and economic conditions. In addition, there is a junior anesthesia student assisting the anesthesia team that is confident but prone to medical error. At the case conclusion, the team must decide how to proceed with critical care when no critical care beds are available. |
| **Primary Learning Objectives** | 1. Recognize and provide a differential diagnosis for the acute abdomen. 2. Recognize severe volume depletion and provide appropriate resuscitation. 3. Communicate effectively and respectfully with team members including those with less training and experience. 4. Weigh ethical considerations for allocation of scarce resources for critically ill patients in a low-resource environment. |
| **Critical Actions** | Surgical trainee:   - Recognize delayed presentation of bowel perforation - Call for anesthesiologist assistance for patient management - Initiate management of sepsis including administration of IV fluids and antibiotics - Communicate effectively with operative team regarding patient acuity and discuss options for postoperative management   Anesthesia trainee:   - Proceed with rapid sequence induction while avoiding worsened hypotension - Prepare norepinephrine infusion to treat septic shock - Recognize student’s level of experience and closely supervise intubation and patient management in a respectful manner - Identify and correct esophageal intubation - Communicate effectively with operative team regarding patient acuity and discuss options for postoperative management |
| **Learner Preparation or Prework** | The surgical resident is called to the OR to assess a patient brought emergently for surgery. The patient is a 10-year-old, 45kg male. He is lethargic and appears very ill with fever, tachycardia and hypotension. An abdominal x-ray showed free air under the diaphragm. He was taken to the operating room because of the expected need for an operation, and consent was obtained from the mother for exploratory laparotomy. |

| Initial Presentation – | | | |
| --- | --- | --- | --- |
| **Initial vital signs** | HR 154, BP 76/42, SpO2 90%, RR 28, temperature 39.2. Mental status: lethargic but responsive, in pain | | |
| **Overall Setting and Appearance** | Patient in operating room, monitors are attached but values only displayed when asked for.  Anesthesia machine is in room with halothane vaporizer, table with vial of thiopental (500mg), suxamethonium, pancuronium, adrenaline (1mg), noradrenaline (1mg), ketamine (50mg), syringes and fluid bags available.  After the patient is induced and intubated the patient will be draped; a laparoscopy or bowel anastomosis simulator is available in room and will be used by the surgery resident to simulate performing an operation in order to complete the scenario.  A nurse anesthesia student is in the room but is very inexperienced and requires close supervision. | | |
| **Actors (e.g., standardized participants) and their roles in the room at case start** | Scrub technician to set-up and assist with surgery.  Student nurse anesthetist to provide information initially to surgeon, then to be the hands-on anesthesia provider supervised by the anesthesia resident. | | |
| **HPI** | The patient is a 10-year-old, 45kg male. He is lethargic and with fever, tachycardia and hypotension. This was preceded by three days of persistent abdominal pain and vomiting.  *Additional information may be provided by anesthetist when asked:*  The mother described that last oral intake was two days ago. He has had very little urine output since, and no bowel movements. His mother says he needed surgery last year after a similar illness, but she doesn’t remember anything more specific. Abdomen is tender, distended, minimal bowel sounds. An abdominal x-ray showed free air under the diaphragm. | | |
| **Past Medical/Surgical History** | **Medications** | **Allergies** | **Family History** |
| Open appendectomy 1 year ago | None | None | None |
| **Physical Examination** | | | |
| **General** | Male child who is lethargic, anxious, clearly in acute distress due to abdominal pain | | |
| **HEENT** | Pupils equal, round and reactive. No lymphadenopathy. | | |
| **Neck** | Range of motion normal. | | |
| **Lungs** | Breath sounds equal and clear bilaterally. Respiratory rate increased. No stridor or wheeze. | | |
| **Cardiovascular** | Tachycardic and hypotensive. Normal S1, S2 with no murmur. 1+ radial, femoral and dorsalis pedis pulses. | | |
| **Abdomen** | Rigid, diffusely tender, distended, minimal bowel sounds. | | |
| **Neurological** | No focal neurologic deficits. Cranial nerves intact and symmetric. Moves all extremities equally. | | |
| **Skin** | Mottled, no rash. | | |
| **GU** | Deferred. | | |
| **Psychiatric** | Anxious, unable to fully assess due to patient distress and lethargy. | | |

| Instructor Notes - Changes and CASE Branch Points | | |
| --- | --- | --- |
| **Intervention / Time point** | **Change in Case** | **Additional Information** |
| Learner (surgical resident) arrives to room and begins initial assessment; asks for vital signs, lab work and/or abdominal films. | Student nurse anesthetists says he is glad the surgeon has arrived, and he thinks this patient needs an abdominal operation.  Patient is lethargic and mostly unresponsive. | Student nurse anesthetist can provide answers to questions about patient evaluation.  Anesthetist may ask if they should prepare for the operation. Assume that family has already provided consent for the operation. |
| Surgeon begins volume resuscitation, antibiotics. Learner agrees that patient requires operative intervention and calls for anesthesia supervisor. | Student asks how much fluid the patient should receive. Fluid endpoint may be based on vital signs or UOP.  Anesthetist calls for supervisor to come to OR, gives report and stays at head of bed preparing for intubation.  HR improves to 130, BP to 90/60 with fluid administration. SpO2 95%. |  |
| Anesthesia trainee asks for report on patient status and history. May request additional fluid administration.  Discusses induction plan with student nurse anesthetist. | Induction with ketamine, thiopental, suxamethonium and/or pancuronium. Trainee listens for breath sounds and looks for chest rise, neither of which are present.  After induction with ketamine, HR to 135, BP to 106/66. SpO2 98%, drops quickly to 90% and 80% if esophageal intubation not recognized.  After induction with thiopental, HR to 145, BP to 66/40. SpO2 98%, drops quickly to 90% and 80% if esophageal intubation not recognized. | If anesthesia trainee is unsure of how to draw up or administer drugs, may ask for assistance from anesthetist.  The student insists on performing the intubation for educational purposes but results in an unrecognized esophageal intubation. If trainee asks for etCO2 tracing it is unavailable because the monitor is broken. |
| Surgeon and scrub tech put on gowns, prepare to drape patient. Laparoscopic or bowel anastomosis simulator is set up and started.  Anesthetist performs intubation after anesthesia induction. Expresses confidence that intubation was successful. Breath sounds are not heard. | Anesthesia trainee recognizes esophageal intubation, intervenes and intubates successfully.  Surgical safety checklist performed. Surgeon begins laparoscopy simulation. |  |
| SpO2 improves to 100% once reintubation is performed.  Surgeon and scrub tech participate in time out. Patient is draped, laparoscopy or bowel anastomosis simulation begins. | BP drifts downward from 100/60 to 80/50 to 72/40. Responsive briefly to adrenaline bolus but then drifts to 60/40. HR increasing from 130 to 150. SpO2 92%. | Scrub tech describes details of operative findings: soiling of abdominal contents, discrete area of bowel ischemia with perforation near ileocecal valve. |
| Anesthesia trainee alerts surgical team to dropping BP, treats accordingly. | BP remains low around 80/50, HR 130 and stabilizes with vasopressor infusion. SpO2 92% despite 100% FiO2. | If vasopressor infusion is requested, trainee must mix infusion and administer without a pump. May ask for assistance from student.  ABG (if requested, and deemed available): pH: 7.18, PaO_2_: 88, PaCO_2_: 34, Lactate: 7.0 |
| Discussion between anesthesia and surgery trainees about ICU admission. Prompted to remain intubated and admit directly to ICU. | Scrub tech states that the operation is near completion. “Where should this child be admitted after?” |  |
| Student contacts the ICU and is told that there are only 3 ventilators. All are being used currently. One patient has HIV and severe pneumonia. Another is a trauma patient planning to be extubated in the morning. The other is a 70yo patient with CHF exacerbation. | Discussion among trainees about how best to proceed |  |

**Ideal Scenario Flow**

The first learner (surgical trainee) will start by evaluating the patient and providing preoperative resuscitation. With stabilization, the decision is made to proceed with surgery. When called for, the second learner (anesthesia trainee) will come to assist with induction and intubation. The surgical trainee will commence with surgery while remaining attentive to the patient status, while the anesthesia trainee manages the student anesthetist and provides ongoing resuscitation.

Providers will need to recognize delayed presentation of surgical disease and provide appropriate and aggressive initial management. Anesthesia trainees will be required to prepare and utilize medications they may not be familiar with for induction and blood pressure management, and also supervise a very inexperienced student prone to making errors (i.e. esophageal intubation). At the conclusion of the procedure the team will need to discuss and decide how to allocate limited resources when there are not enough ventilators available for all of the critically ill patients.

**Anticipated Management Mistakes**

1. Failure to move through case efficiently: The teaching points for this case require progression from assessment to management of a septic patient over a compressed timeframe. Facilitators will need to guide actors to communicate case progression at several steps including the need to proceed with the operation and the need to communicate postoperative planning to the ICU.
2. Trainees focused exclusively on medical management: As advanced residents, the trainees are expected to manage the basics of resuscitation and the goal of the scenario is for the them to communicate and co-manage the patient while remaining attentive to other tasks. For the surgical trainee, the distraction is a technical task of performing a bowel anastomosis simulation. For the anesthesia trainee, the distraction is supervising an inexperienced anesthesia student.
3. Failure to effectively supervise and correct anesthesia student: In addition to clinical care, this scenario introduces learners to a challenging supervisory situation involving an inexperienced trainee who makes a medical error. Identifying and correcting an error in a respectful and effective way is difficult in any context but is made more challenging by the communication and cultural barriers introduced in a non-native setting.
4. Inability to make a decision about patient disposition: The key resource limitation in this case occurs at the end when the critically ill patient requires admission to an intensive care unit where there are no available ventilators. Learners should be encouraged to consider what factors to include in making this decision, or to be creative in looking for alternative options, while maintaining an appropriate cultural stance as a visitor.
