# Supplementary material for: The Preparing Residents for International Medical Experiences (PRIME) Simulation Workshop: Equipping Surgery and Anesthesia Trainees for International Rotations
Source: MedEdPORTAL. 2021 Feb 11;17:11088. doi: 10.15766/mep_2374-8265.11088 (PMC7880254; doi:10.15766/mep_2374-8265.11088)
Supplement: Supplementary file 1 — Simulation 1.docxSimulation 2.docxSimulation 3.docxSimulation 2 Lab Values.docxSimulation 3 Lab Values.docxResident Self-Assessment.docxCritical Actions Checklist.docxDebriefing Guide.docxSimulation Evaluation.docx [file mep_2374-8265.11088-s001.zip › C. Simulation 3.docx]

| **Appendix C: MedEdPORTAL Simulation Case 3**  SIMULATION CASE TITLE: Eclampsia in a low-resource setting  AUTHORS: J. Matthew Kynes, MD, Rondi Kauffmann, MD, Arna Banerjee, MD, Chris Sizemore, MD  LEARNER AUDIENCE: Residents in obstetrics/gynecology, general surgery and anesthesiology | |
| --- | --- |
| **PATIENT NAME:** Mary Mungai  **PATIENT AGE:** 22 years old  **CHIEF COMPLAINT:** Headache, elevated blood pressure in pregnancy  **PHYSICAL SETTING:** The scenario begins in a patient examination room and then progresses to the operating room | |
|  | |
| **Brief narrative description of case** | The obstetric resident is called by a nurse to evaluate a new patient. Per the nurse, she is a 22-year-old female primigravid at an advanced stage of pregnancy with no prior prenatal care. No other history is available, and the patient speaks only a distant tribal dialect. The patient’s vital signs, lab work and eventual seizure indicate an episode of eclampsia which must be stabilized and progress quickly to delivery. Delivery is complicated by postpartum hemorrhage and neonatal distress that requires active communication among trainees for successful management.  Participants will be forced to make clinical decisions based on limited information which may be more common in a low-resource setting. In addition, they must determine how to allocate human resources to effectively care for a mother and neonate in distress at the same time. |
| **Primary Learning Objectives** | 1. Identify and manage preeclampsia/eclampsia in a parturient with a language barrier and limited staff support 2. Induce anesthesia safely for an urgent c-section using drugs common in an international setting, understanding indications and contraindications for general vs. neuraxial anesthesia 3. Perform safe c-section and manage related complications including post-partum hemorrhage and neonatal resuscitation with consideration for scarce resources in an international setting 4. Recognize clinical or social situations that are beyond area of expertise or experience and know when to defer to other providers 5. Demonstrate effective interdisciplinary communication, teamwork and leadership skills |
| **Critical Actions** | Obstetric trainee:   - Identify preeclampsia/eclampsia using clinical clues and initiate blood pressure management - Obtain clinical information while unable communicate effectively with a patient due to a language barrier - Determine need to proceed with delivery with advanced eclampsia - Report key clinical information to anesthesia team for effective anesthesia planning - Communicate technical instructions for c-section to an assistant with limited experience - Discuss plan for hemorrhage management with limited blood transfusion capabilities   Anesthesia trainee:   - Identify preeclampsia/eclampsia using clinical clues and formulate appropriate anesthesia plan - Treat elevated blood pressure with antihypertensive - Communicate with obstetric team regarding amount of hemorrhage and treat with first and second line uterotonic - Discuss plan for hemorrhage management with limited blood transfusion capabilities - Initiate neonatal resuscitation by assessing patient and beginning with effective rescue breathing - Call for help or assign alternative provider to assist with maternal care during neonatal resuscitation |
| **Learner Preparation or Prework** | You are the obstetrician called by a nurse to evaluate a new patient. Per the nurse, she is a 22-year-old female primigravid at an advanced stage of pregnancy with no prior prenatal care. No other history is available, and the patient speaks only a distant tribal dialect. |

| Initial Presentation – | | | |
| --- | --- | --- | --- |
| **Initial vital signs** | HR 102, BP 178/120, SpO2 94%, RR 22. Mental status: Anxious, in distress, speaking in a foreign language. | | |
| **Overall Setting and Appearance** | Clinical domain at initiation of simulation is the L&D ward (OR with curtain pulled to hide anesthesia machine and cart). SimMom with gravid uterus, when spoken to only responds in Swahili. Upon decision to proceed to OR, curtain can be withdrawn or team brought to adjacent operating room and all OR equipment necessary for c-section is made available. An isolette with a neonatal model and resuscitation equipment should be available. | | |
| **Actors (e.g., standardized participants) and their roles in the room at case start** | The first actor to arrive is an obstetric intern who will provide additional clinical information on the mother as an interpreter. This actor will also prompt the obstetrician to proceed to surgery.  In the operating room, there is a nurse to facilitate surgery, reveal postpartum hemorrhage and provide initial assessment of the neonate after delivery. In addition, there will be an anesthetist who will provide handover to the arriving anesthesia trainee at the beginning of the operation. | | |
| **HPI** | The patient is a 22-year-old female primigravid at an advanced stage of pregnancy with no prior prenatal care. No other history is available, and the patient speaks only a distant tribal dialect.  After the interpreter arrives, it is determined that she has had a prior c-section and that her headaches have progressed over several days. She also has blurred vision and right upper quadrant abdominal pain. There has been no vaginal bleeding or contractions, and she continues to feel fetal movement. | | |
| **Past Medical/Surgical History** | **Medications** | **Allergies** | **Family History** |
| Prior c-section x1 | None | None | None |
| **Physical Examination** | | | |
| **General** | Parturient who is anxious due to headache and concern for her pregnancy, in acute distress due to headache and right upper quadrant pain. Frequently reaches to head and right upper quadrant | | |
| **HEENT** | Pupils equal, round and reactive. No lymphadenopathy | | |
| **Neck** | Range of motion normal and supple | | |
| **Lungs** | Breath sounds equal and clear bilaterally. Respiratory rate increased. No stridor or wheeze | | |
| **Cardiovascular** | Tachycardic and hypertensive. Normal S1, S2 with no murmur. 2+ radial, femoral and dorsalis pedis pulses. | | |
| **Abdomen** | Gravid consistent with 34 week gestation, tender to right upper quadrant, not distended, bowel sounds present | | |
| **Neurological** | No focal neurologic deficits. Cranial nerves intact and symmetric. Moves all extremities equally. When asked, visual fields are blurry | | |
| **Skin** | No rash. 2+ edema to lower extremities bilaterally | | |
| **GU** | Deferred | | |
| **Psychiatric** | Anxious, unable to fully assess due to language barrier | | |

| Instructor Notes - Changes and CASE Branch Points | | |
| --- | --- | --- |
| **Intervention / Time point** | **Change in Case** | **Additional Information** |
| Trainee greets patient and begins history and physical exam. | Display initial vital signs in response to placement of monitors by trainee.  Patient moans intermittently. | Patient speaks only in Swahili. Interpreter is not available until the OB intern arrives later in the script.  Monitors may be placed at any time.  Trainee should work to establish IV access. |
| Repeat BP increased to 192/122  Asks for or takes samples for lab – blood and urine (if allowed at instructor’s discretion) | Treats BP with labetalol or hydralazine. Nitroglycerine, nicardipine are not available.  Decrease BP on next check after treatment to 168/104. If no treatment administered, increase BP to 202/124.  Decrease SpO2 slowly to 90%.  When nurse returns, asks for OB consultation. | If lab work has not been asked for or obtained by trainee, nurse may prompt by asking trainee if labs are desired. |
| Places nasal cannula or face mask. | Increase SpO2 to 98% |  |
| Initiate seizure.  BP 160/102, HR 106  SpO2 drops to 88%, increases to 96% with supplemental oxygen. | Patient begins to seize, is unresponsive and after seizure is stopped remains very lethargic.  Trainee provides supportive measures (O2, suctions airway, turns patient to side)  Trainee gives valium to stop seizure, gives magnesium sulfate in IV or IM dose | Trainee may request anesthesia or surgical help at anytime. |
| ‘OB intern’ arrives as seizure is treated, states her role. Asks if she can translate for the patient.  Translation for patient reveals that she believes she is 34 weeks pregnant. She was complaining of a headache, and reportedly had an episode of blurry vision this morning. | Trainee decides to proceed to operating room and asks for anesthesia trainee, if not present already.  If asks for lab work to make decision on anesthetic technique this can be provided at this time. | OB intern states that the patient needs to go to theater. Asks, “Should I prepare the room for GA or spinal?”  Brief pause to either pull back curtain to reveal theater or move from L&D ward to theater setup |
| PART II: OPERATING ROOM |  |  |
| Scenario transitions to theater with patient on the table and intubated. ‘OB intern’ states that they need to get the baby out urgently  Patient is already induced and intubated  Scrub tech and OB intern are preparing patient for incision | Anesthesia resident receives handoff from anesthetist who has been working for 24 hours and is off duty.  Scrub tech and OB intern are preparing patient for incision.  BP 108/72, HR 108, SpO2 100%, RR coordinated to ventilator setting. | Trainees should request pediatrics consultation at any point. Nurse informs them that the pediatrician will not return to the hospital for two days.  Anesthesia trainee may request additional anesthesia help.  Only halothane available for maintenance. N2O is not available. |
| With initiation of halothane patient has occasional PVCs on ECG.  C-section proceeds with prompting by scrub tech. A surgical trainee arrives to the room to assist with the operation and may receive instruction from the obstetric trainee. | BP 108/72, HR 108, SpO2 100%, RR coordinated to ventilator setting.  Obstetric and surgical trainees proceed with c-section. |  |
| Baby is delivered and brought to isolette by nurse.  After delivery, intern states that there is quite a lot of blood and that the uterus is boggy.  If blood administration is not mentioned then intern may ask if the trainees think the patient will need a transfusion. | Patient BP 80/50, HR 130.  With epinephrine, BP improves briefly to 106/60, HR to 135.  Anesthesia resident gives oxytocin into IV fluids to administer. May treat low BP with adrenaline which is the only pressor available. May proceed with IM ergometrine (misoprostol is not available).  If surgical resident or anesthesia resident asks for blood transfusion availability, they are told that someone needs to be called to donate it. | Circulating RN should reveal suction canister filled with blood indicating large amount of bleeding.  There are no units available in the blood bank. In order to give blood, someone will need to be called in to donate. Residents must decide if they will call someone in for donation. |
| Baby is in isolette and nurse asks for help. Baby is not breathing well and has meconium staining | Mother VS: BP 80/50, HR 130  Newborn has HR 120, gasping respiratory pattern. SpO2 not available.  Anesthesia resident and surgery resident must decide how to divide management between postpartum hemorrhage and newborn in distress. Help not immediately available.  Resident responding to neonate should give rescue breaths. | RN should stay with neonate and give clues to patient status (gasping initially, HR 120). |
| With effective rescue breaths, HR improves initially but then drops to 50 requiring chest compressions. After 1 minute of chest compression, resident is told that HR has improved. | Neonate management is ongoing. Resident attending to neonate should start chest compressions when HR drops to 50, after 1 minute HR can return to normal with good respiratory effort.  Maternal management of hypotension and hemorrhage, uterus is soft until 2^nd^ line agent is given. Bleeding continues until decision to call for blood donation  Maternal BP 80-90/50-60 and HR 120-130 with bleeding. | Simulation complete when neonatal resuscitation is performed and decision to transfuse mother. |

**Ideal Scenario Flow**

The parturient is non-English speaking and presents with advanced eclampsia. The treating provider must diagnose and provide initial management with limited assistance for additional staff and decide to proceed to operating room for delivery. Clinical clues must be obtained without the ability to communicate effectively with the patient initially. Eventually, the obstetric intern actor will provide interpretation services to facilitate diagnosis and progress with the case to c-section.

An additional anesthesia and surgery trainee have been waiting for the second part of the scenario. In the operating room, the patient is already under general anesthesia and surgery is about to begin. The anesthesia trainee will receive report from the obstetric trainee and an actor anesthetist. The obstetric trainee will be asked to perform the c-section with the assistance of a surgery trainee who may be inexperienced or unfamiliar with the procedure. The team will also need to manage post-partum hemorrhage and decide on blood transfusion in a setting where additional effort is required to obtain it when a blood bank is not available. Finally, the neonate will be in distress and the trainees must communicate about providing neonatal resuscitation while also caring for the mother.

**Anticipated Management Mistakes**

1. Obstetric trainee fails to diagnose eclampsia due to language barrier: The patient’s language barrier and lack of interpreter initially may be a significant distractor for the trainee despite the presence of hypertension, headache and seizure. In this case, the actor may enter earlier to prompt the trainee to the correct diagnosis and allow the case to proceed.
2. A foreign language speaker is unavailable to speak for the patient: Some institutions may have a foreign language speaker available, ideally in the language likely to be encountered at the rotation site. However, a pre-recorded selection of foreign language speaking may also be used.
3. Anesthesia trainee fails to initiate neonatal resuscitation or leaves surgical patient unattended: This is a key management point in the scenario but one in which the trainee may feel ill-prepared to handle. If either patient is left unattended, this should be brought up in the debriefing as an opportunity to consider human resource allocation and the need to be flexible when clinical personnel are scarce. This may also occur from trainee unfamiliarity with neonatal resuscitation protocols, in which case the nurse actor may provide prompting to guide initial steps of the resuscitation process.
