# Supplementary material for: The Preparing Residents for International Medical Experiences (PRIME) Simulation Workshop: Equipping Surgery and Anesthesia Trainees for International Rotations
Source: MedEdPORTAL. 2021 Feb 11;17:11088. doi: 10.15766/mep_2374-8265.11088 (PMC7880254; doi:10.15766/mep_2374-8265.11088)
Supplement: Supplementary file 1 — Simulation 1.docxSimulation 2.docxSimulation 3.docxSimulation 2 Lab Values.docxSimulation 3 Lab Values.docxResident Self-Assessment.docxCritical Actions Checklist.docxDebriefing Guide.docxSimulation Evaluation.docx [file mep_2374-8265.11088-s001.zip › D. Simulation 2 Lab Values.docx]

**Appendix D. Lab Values for Simulation 2**

**Lab Values and Studies (provided when asked):**

WBC: 22.1, HGB: 17.3, PLT: 475,000

Type and screen: O +, negative antibody screen

Na: 128, K: 2.4, Cl: 92, HCO3: 17, BUN: 72, Creat: 2.2, Glucose: 98

AST: 154, ALT 122, LDH: 320

pH: 7.22, PaO_2_: 92, PaCO_2_: 34, Lactate: 8.0

PT: 12 (12-14), PTT 23 (22-28), INR 1.1

Abdominal plain films, flat and upright: Dilated small-bowel loops with air fluid levels indicate SBO. Free air present under the diaphragm.


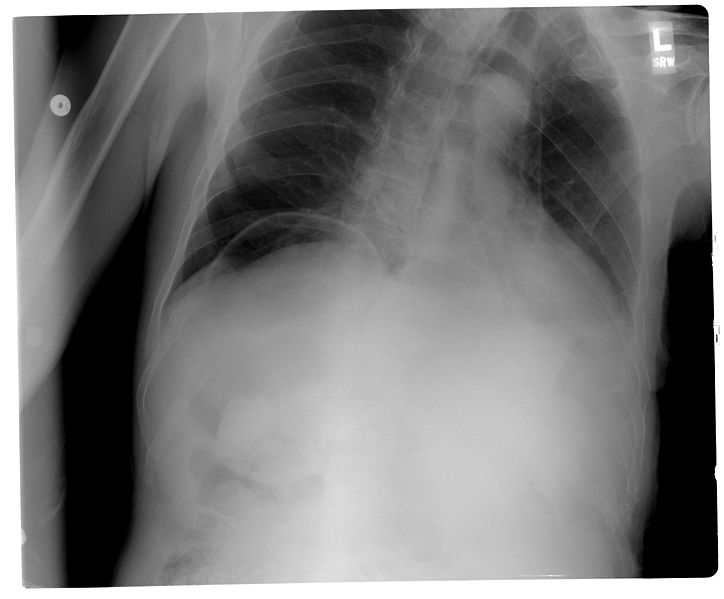


<https://commons.wikimedia.org/wiki/File:Pneumoperitoneum_chest_X-ray.jpg>, Licensed for distribution Creative Commons 2.0 (attribution only)
