# Supplementary material for: The Preparing Residents for International Medical Experiences (PRIME) Simulation Workshop: Equipping Surgery and Anesthesia Trainees for International Rotations
Source: MedEdPORTAL. 2021 Feb 11;17:11088. doi: 10.15766/mep_2374-8265.11088 (PMC7880254; doi:10.15766/mep_2374-8265.11088)
Supplement: Supplementary file 1 — Simulation 1.docxSimulation 2.docxSimulation 3.docxSimulation 2 Lab Values.docxSimulation 3 Lab Values.docxResident Self-Assessment.docxCritical Actions Checklist.docxDebriefing Guide.docxSimulation Evaluation.docx [file mep_2374-8265.11088-s001.zip › E. Simulation 3 Lab Values.docx]

Appendix E. Simulation 3 Lab Values.

*Lab results may be made available at instructor’s discretion.*

WBC: 7.4, HB: 11.3, PLT: 34,000

Type and screen: A -, negative antibody screen

BMP: not available; Glucose 11.5mmol/L

Total protein: 7.0, Albumin: 3.3, AST: 56, ALT 72, LDH: 220

PT: 14 (12-14), PTT 29 (22-28), INR 1.2

Urine dipstick: 4+ protein, 1+ glucose
