# Supplementary material for: The Preparing Residents for International Medical Experiences (PRIME) Simulation Workshop: Equipping Surgery and Anesthesia Trainees for International Rotations
Source: MedEdPORTAL. 2021 Feb 11;17:11088. doi: 10.15766/mep_2374-8265.11088 (PMC7880254; doi:10.15766/mep_2374-8265.11088)
Supplement: Supplementary file 1 — Simulation 1.docxSimulation 2.docxSimulation 3.docxSimulation 2 Lab Values.docxSimulation 3 Lab Values.docxResident Self-Assessment.docxCritical Actions Checklist.docxDebriefing Guide.docxSimulation Evaluation.docx [file mep_2374-8265.11088-s001.zip › F. Resident Self-Assessment.docx]

Appendix F. Resident Self-Assessment

Survey Questions Part 1

To the best of your ability, please rate yourself on according to the following scale for each of the scenarios provided:

*“I feel confident to participate in this activity…”*

1. Exploratory laparotomy for a patient with sepsis from intestinal perforation when invasive monitoring is not available:

1. Only as an observer

2. With direct supervision

3. With indirect supervision

4. Independently

5. As an instructor of junior colleagues

1. Triage and stabilization of multiple trauma patients with life-threatening injuries and one available operating room:

1. Only as an observer

2. With direct supervision

3. With indirect supervision

4. Independently

5. As an instructor of junior colleagues

1. General anesthesia for thoracotomy and hemorrhage control with central oxygen and power failure:

1. Only as an observer

2. With direct supervision

3. With indirect supervision

4. Independently

5. As an instructor of junior colleagues

1. Discussion of an accidental intrathecal injection of TXA by a junior trainee under your supervision:

1. Only as an observer

2. With direct supervision

3. With indirect supervision

4. Independently

5. As an instructor of junior colleagues

1. Decision-making regarding post-operative care of postpartum patient in pulmonary edema following hemorrhage with all available ICU ventilators occupied:

1. Only as an observer

2. With direct supervision

3. With indirect supervision

4. Independently

5. As an instructor of junior colleagues

Survey Questions Part 2

To the best of your ability, please rate yourself on a scale of 1 to 5 for each of the following competencies applied in a low-resource setting (i.e. at Kijabe Hospital). Level one is a beginning resident; Level 2 is beyond introductory but below a mid-level resident; Level 3 is a mid-level resident; Level 4 is a graduating resident or fellow; Level 5 exceeds expectations of a resident.

*Patient care*

| Pre-anesthetic evaluation and preparation | | | | | |
| --- | --- | --- | --- | --- | --- |
| Has not achieved Level 1 | Level 1 | Level 2 | Level 3 | Level 4 | Level 5 |
|  | Performs general histories and physical examinations  Identifies clinical issues relevant to anesthetic care with direct supervision  Identifies the elements and process of informed consent | Identifies disease processes and medical issues relevant to anesthetic care  Optimizes preparation of non-complex patients receiving anesthetic care  Obtains informed consent for routine anesthetic care; discusses likely risks, benefits, and alternatives in a straightforward manner; responds appropriately to patient’s or surrogate’s questions; recognizes when assistance is needed | Identifies disease processes and medical or surgical issues relevant to subspecialty anesthetic care; may need guidance in identifying unusual clinical problems and their implications for anesthesia care  Optimizes preparation of patients with complex problems or requiring subspecialty anesthesia care with indirect supervision  Obtains appropriate informed consent tailored to subspecialty care or complicated clinical situations with indirect supervision | Performs assessment of complex or critically-ill patients without missing major issues that impact anesthesia care with conditional independence  Optimizes preparation of complex or critically- ill patients with conditional independence  Obtains appropriate informed consent tailored to subspecialty care or complicated clinical situations with conditional independence | Independently performs comprehensive assessment for all patients  Independently serves as a consultant to other members of the health care team regarding optimal pre-anesthetic preparation  Consistently ensures that informed consent is comprehensive and addresses patient and family needs |

| Peri-anesthetic complication management | | | | | |
| --- | --- | --- | --- | --- | --- |
| Has not achieved Level 1 | Level 1 | Level 2 | Level 3 | Level 4 | Level 5 |
|  | Performs patient assessments and identifies complications associated with patient care; begins initial management of complications with direct supervision | Performs post-anesthetic assessment to identify complications of anesthetic care; begins initial management of peri- anesthetic complications with direct supervision | Identifies and manages peri-anesthetic complications unique to subspecialty or medically complex patients, and requests appropriate consultations with indirect supervision | Identifies and manages all peri- anesthetic complications with conditional independence | Independently identifies and manages all peri- anesthetic complications |

| Crisis management | | | | | |
| --- | --- | --- | --- | --- | --- |
| Has not achieved Level 1 | Level 1 | Level 2 | Level 3 | Level 4 | Level 5 |
|  | Recognizes acutely ill or medically deteriorating patients; initiates basic medical care for common acute events; calls for help appropriately | Constructs prioritized differential diagnoses that include the most likely etiologies for acute clinical deterioration; initiates treatment with indirect supervision and seeks direct supervision appropriately | Identifies and manages clinical crises with indirect supervision; may require direct supervision in complex situations | Identifies and manages clinical crises appropriately with conditional independence; assumes increasing responsibility for leadership of crisis response team | Coordinates crisis team response |

| Triage of critically-ill patient in non-operative setting | | | | | |
| --- | --- | --- | --- | --- | --- |
| Has not achieved Level 1 | Level 1 | Level 2 | Level 3 | Level 4 | Level 5 |
|  | Performs a focused evaluation of the critically-ill patient; monitors patient’s clinical status to identify acute changes and trends; communicates pertinent findings to supervisor  Participates in development and initiation of a treatment plan as directed by supervisor | Identifies relevant critical disease processes requiring urgent or emergent intervention; seeks assistance to identify appropriate care setting (e.g., ICU, transitional care unit)  Develops, implements, and appropriately modifies treatment plan based on patient’s response with direct supervision | Identifies appropriate care setting and coordinates patient’s disposition with direct supervision  Prioritizes clinical management of clinical problems with indirect supervision | Identifies appropriate care setting and coordinates patient’s disposition with indirect supervision  Defines clinically appropriate priorities when resources are limited  Integrates management choices taking into account long-term impact of therapeutic decisions with indirect supervision  Supervises other members of the health care team | Coordinates transition of care to appropriate care setting; sets clinically appropriate priorities when resources are limited  Serves as a consultant to other members of the health care team regarding initial evaluation and management of the critically-ill patient |

*Practice-based learning and improvement*

| Education of patients, families, students, residents and other health professionals | | | | | |
| --- | --- | --- | --- | --- | --- |
| Has not achieved Level 1 | Level 1 | Level 2 | Level 3 | Level 4 | Level 5 |
|  | Discusses medical plans and responds to questions from patients and their families  Acknowledges limits and seeks assistance from supervisor | Explains anesthetic care to patients and their families  Teaches basic anesthesia concepts to students and other health care professionals | Effectively explains subspecialty anesthetic care to patients and their families  Teaches anesthesia concepts to students and other residents | Explains anesthesia care and risk to patients and their families with conditional independence  Teaches anesthesia concepts, including subspecialty care, to students, other residents, and other health professionals | Serves as an expert on anesthesiology to patients, their families, and other health care professionals, (locally or nationally)  Participates in community education about anesthesiology |

*Professionalism*

| Responsibility to maintain personal, emotional, physical and mental health | | | | | |
| --- | --- | --- | --- | --- | --- |
| Has not achieved Level 1 | Level 1 | Level 2 | Level 3 | Level 4 | Level 5 |
|  | Demonstrates basic professional responsibilities, such as reporting for work rested and prepared, with appropriate professional attire and grooming  Demonstrates knowledge of basic requirements related to fatigue management, sleep deprivation, and principles of physician well-being  Recognizes the need to balance patient, personal, institutional, and societal needs when providing health care  Complies with training on physician impairment  Identifies departmental and institutional resources available for assistance with concerns about an impaired health care provider | Complies with requirements to assist with preservation of health and mitigation of fatigue (e.g., work hours rules)  Demonstrates the ability to balance personal, institutional, and societal goals with professional responsibilities  Complies with systems intended to prevent physician impairment, (e.g., controlled substance policies) | Reports concerns about the health or well-being of colleagues to a more experienced individual | Reinforces to junior colleagues the importance of compliance with systems to prevent impairment | Serves as a resource for the development of organizational policies and procedures regarding professional responsibilities  Serves as a resource for the development of institutional policies on work-life balance  Serves as a resource for the development of organizational policies and procedures for impaired physicians  Assists with or leads management of suspected impaired colleagues Serves as monitor/resource for colleagues returning from treatment for impairment |

| Responsibility to patients, families and society | | | | | |
| --- | --- | --- | --- | --- | --- |
| Has not achieved Level 1 | Level 1 | Level 2 | Level 3 | Level 4 | Level 5 |
|  | Acts responsibly and reliably with commitment to patient care as expected for level of experience  Completes most assigned clinical tasks on time, but may occasionally require direct supervision  Recognizes a patient’s right to confidentiality, privacy, and autonomy, and treats patients and their families with compassion and respect  Seeks assistance appropriate to the needs of the clinical situation while taking into consideration one's own experience and knowledge  Displays sensitivity and respect for the needs of diverse patient populations and challenges associated with limited access to health care | Completes routine tasks reliably in uncomplicated circumstances with indirect supervision  Identifies issues of importance to diverse patient populations and how limited resources may impact patient care and resource allocation | Completes tasks reliably in complex clinical situations or unfamiliar environments, utilizing available resources, with indirect supervision  Identifies options to address issues of importance to diverse patient populations, and creates strategies to provide care when patient access or resources are limited | Completes all work assignments reliably and supports other providers to ensure patient care is optimized; supervises and advises junior residents on time and task management with conditional independence | Manages the health care team to ensure patient care is the first priority while considering the needs of team members  Completes all work assignments reliably, and independently supports other providers to ensure patient care is optimized  Demonstrates leadership in managing multiple competing tasks  Manages the health care team in a manner that is respectful of patient confidentiality, privacy, and autonomy, and ensures that patients and their families are treated with compassion and respect  Demonstrates mentorship and role modeling regarding responsibilities to diverse patient populations and optimizing patient care when resources are limited |

*Interpersonal and communication skills*

| Team and leadership skills | | | | | |
| --- | --- | --- | --- | --- | --- |
| Has not achieved Level 1 | Level 1 | Level 2 | Level 3 | Level 4 | Level 5 |
|  | Recognizes and respects the expertise of other members of the health care team  Functions effectively as a member of the health care team | Identifies the care team member with appropriate expertise to address a clinical issue  Participates actively in team-based conferences or meetings related to patient care | Coordinates team- based care in routine circumstances | Demonstrates leadership skills in relationships with members of the anesthesia and other patient care teams  Facilitates team-based conferences or meetings related to patient care | Effectively contributes to and leads team-based decision making and clinical care  Participates in and provides leadership in the practice of team-based care |

| Communication with other professionals | | | | | |
| --- | --- | --- | --- | --- | --- |
| Has not achieved Level 1 | Level 1 | Level 2 | Level 3 | Level 4 | Level 5 |
|  | Communicates effectively and with respect for the skills and contributions of other members of the health care team  Identifies interpersonal conflicts and ineffective communication with other members of the health care team, and participates in their resolution as appropriate to level of education  Communicates patient status to supervisors and other providers effectively, including during hand-offs and transitions of patient care  Provides legible, accurate, complete, and timely documentation in written and electronic forms  Respects patient privacy in all environments  Identifies and discloses medical errors or complications to the healthcare team | Identifies institutional resources to assist in conflict resolution  Effectively communicates relevant patient issues during transitions or transfers of care  Uses the medical record to document medical decision making and facilitate patient care  Documentation is clear and concise, addressing key issues relevant to the care of the patient | Adapts communication to the unique circumstances, such as crisis management and subspecialty anesthesia care  Uses institutional resources to assist in conflict resolution | Communicates effectively in crises and contentious situations  Participates in conflict resolution with conditional independence | Mentors other members of the health care team to improve communication skills  Effectively manages conflict in all situations |
