# Supplementary material for: The Preparing Residents for International Medical Experiences (PRIME) Simulation Workshop: Equipping Surgery and Anesthesia Trainees for International Rotations
Source: MedEdPORTAL. 2021 Feb 11;17:11088. doi: 10.15766/mep_2374-8265.11088 (PMC7880254; doi:10.15766/mep_2374-8265.11088)
Supplement: Supplementary file 1 — Simulation 1.docxSimulation 2.docxSimulation 3.docxSimulation 2 Lab Values.docxSimulation 3 Lab Values.docxResident Self-Assessment.docxCritical Actions Checklist.docxDebriefing Guide.docxSimulation Evaluation.docx [file mep_2374-8265.11088-s001.zip › G. Critical Actions Checklist.docx]

**Case 1 – Triage for Multiple Trauma in a Low-Resource Emergency Room**

| **Critical Actions** | **Meets Expectations** | **Partially Complete/ With Prompting** | **Below Expectations** | **Comments** |
| --- | --- | --- | --- | --- |
| **Overall** | | | | |
| Divide into teams to assess multiple patients in parallel |  |  |  |  |
| Allocate team members based on patient acuity |  |  |  |  |
| Reallocate human resources when the clinical scenario changes with arrival of a new patient |  |  |  |  |
| Adapt to resource limitations by sharing equipment and monitors, when necessary |  |  |  |  |
| Mobilize resources and communicate to continue to provide care during an unexpected power outage |  |  |  |  |
| **Patient 1** | | | | |
| Assign roles for initial patient assessment and treatment |  |  |  |  |
| Perform a primary survey (A-B-C-D-E) with intervention provided, when appropriate |  |  |  |  |
| Identify hypotension and administer IV fluid bolus |  |  |  |  |
| Identify deteriorating neurologic status after head injury and secure airway |  |  |  |  |
| Perform a secondary survey after primary survey is complete |  |  |  |  |
| Check equipment prior to intubation attempt |  |  |  |  |
| **Patient 2** | | | | |
| Assign roles for initial patient assessment and treatment |  |  |  |  |
| Perform a primary survey (A-B-C-D-E) with intervention provided, when appropriate |  |  |  |  |
| Recognize progressive tachypnea and hemodynamic collapse from tension pneumothorax |  |  |  |  |
| Provide appropriate chest decompression for tension pneumothorax |  |  |  |  |
| Perform a secondary survey after primary survey is complete |  |  |  |  |
| **Patient 3** | | | | |
| Communicate about how to reallocate staff and resources to assess and treat the new arrival |  |  |  |  |
| Assign roles and a perform primary survey (A-B-C-D-E) |  |  |  |  |
| Recognize severe inhalation injury and need for early airway intervention |  |  |  |  |

**Simulation 2 - Delayed presentation of intestinal obstruction**

| **Critical Action** | **Meets Expectations** | **Partially Complete/ With Prompting** | **Below Expectations** | **Comments** |
| --- | --- | --- | --- | --- |
| **Surgical Trainee** | | | | |
| Recognize delayed presentation of bowel perforation |  |  |  |  |
| Call for anesthesiologist assistance for patient management |  |  |  |  |
| Initiate management of sepsis including administration of IV fluids and antibiotics |  |  |  |  |
| Communicate effectively with operative team regarding patient acuity and discuss options for postoperative management |  |  |  |  |
| **Anesthesia Trainee** | | | | |
| Accurately diagnose septic shock |  |  |  |  |
| Proceed with rapid sequence induction using agents to avoid worsened hypotension |  |  |  |  |
| Prepare norepinephrine infusion to treat septic shock |  |  |  |  |
| Recognize student’s level of experience and closely supervise intubation and patient management in a safe and respectful manner |  |  |  |  |
| Identify and correct esophageal intubation |  |  |  |  |
| Communicate effectively with operative team regarding patient acuity and discuss options for postoperative management |  |  |  |  |

**Simulation 3 – Eclampsia in a low-resource setting**

| **Critical Action** | **Meets Expectations** | **Partially Complete/ With Prompting** | **Below Expectations** | **Comments** |
| --- | --- | --- | --- | --- |
| **Obstetric Trainee** |  | | | |
| Identify preeclampsia/eclampsia using clinical clues and initiate blood pressure and seizure management |  |  |  |  |
| Obtain clinical information while unable communicate effectively with a patient due to a language barrier |  |  |  |  |
| Determine need to proceed with delivery with advanced eclampsia |  |  |  |  |
| Report key clinical information to anesthesia team for effective anesthesia planning |  |  |  |  |
| Communicate technical instructions for c-section to an assistant with limited experience |  |  |  |  |
| Discuss plan for hemorrhage management with limited blood transfusion capabilities |  |  |  |  |
| **Anesthesia Trainee** |  | | | |
| Identify preeclampsia/eclampsia using clinical clues and formulate appropriate anesthesia plan |  |  |  |  |
| Treat elevated blood pressure with antihypertensive |  |  |  |  |
| Communicate with obstetric team regarding amount of hemorrhage and treat with first and second line uterotonic |  |  |  |  |
| Discuss plan for hemorrhage management with limited blood transfusion capabilities |  |  |  |  |
| Initiate neonatal resuscitation by assessing patient and beginning with effective rescue breathing |  |  |  |  |
| Call for help or assign alternative provider to assist with maternal care during neonatal resuscitation |  |  |  |  |
