# Supplementary material for: The Preparing Residents for International Medical Experiences (PRIME) Simulation Workshop: Equipping Surgery and Anesthesia Trainees for International Rotations
Source: MedEdPORTAL. 2021 Feb 11;17:11088. doi: 10.15766/mep_2374-8265.11088 (PMC7880254; doi:10.15766/mep_2374-8265.11088)
Supplement: Supplementary file 1 — Simulation 1.docxSimulation 2.docxSimulation 3.docxSimulation 2 Lab Values.docxSimulation 3 Lab Values.docxResident Self-Assessment.docxCritical Actions Checklist.docxDebriefing Guide.docxSimulation Evaluation.docx [file mep_2374-8265.11088-s001.zip › H. Debriefing Guide.docx]

**Appendix H. Facilitator Debriefing Guide**

**Course Learning Objectives**

1. Improve clinical management of scenarios encountered in low-resource settings such as triage of critically-ill patients in a non-operative setting, crisis situations, peri-operative complications with limited diagnostic and therapeutic options, pre-anesthetic and pre-surgical evaluation with limited diagnostic options. (Patient care)
2. Improve knowledge of biomedical and social-behavioral factors affecting clinical outcomes for patients experience poverty and endemic infectious disease. (Medical knowledge)
3. Improve practice-based learning by gaining skills for educating students and other health professionals. (Practice-based learning and improvement)
4. Identify and overcome barriers to personal, emotional, physical and mental health in a high-stress, unfamiliar environment. (Professionalism)

**Common Debriefing Questions**

What do you think you and the team did well during this case?

What did you find challenging about this case?

What in this case differed from clinical care that would be provided at a hospital in a high-income setting?

How did human or material resource availability affect your ability to provide care in this case?

What social, economic, or cultural factors influenced the presentation or management of this patient?

**Scenario-Specific Debriefing Points**

**Simulation 1: Triage of multiple trauma patients in a low-resource emergency room**

The following teaching points may be used for debriefing following this scenario:

- Rapid recognition and stabilization of critical injuries using a systematic approach (A-B-C-D-E) is a key tenet of acute trauma management.
- Effective communication with patients and team members in crisis situations requires clear direction, closed loops, and assignment of roles and responsibilities.
- During a dynamic clinical situation, the organized redeployment of resources based on patient acuity and need may become necessary.
- In any clinical setting, checking equipment before a procedure and having a back-up plan for faulty equipment is a key step to prevent complications from device failure.
- Decision-making for triage of limited resources such as staff or monitors for multiple patients should be based on a rapid assessment of resource availability, personnel skill, and clinical need.
- Even in a major technical failure, such as central electric supply, teams should remain organized and look for alternate ways to continue to provide effective care.

**Simulation 2: Delayed presentation of intestinal obstruction**

The following teaching points may be used for debriefing following this scenario:

- There are multiple reasons for delayed presentation in a low-resource environment (distance, poverty, transportation issues, alternative/traditional healers, misdiagnosis, etc.).
- Delayed presentation may require additional resuscitation prior to beginning surgery to avoid further deterioration with general anesthesia and surgical stress.
- OR staffing may be limited, including pharmacists and assistants for equipment set-up and drug preparation, which requires familiarity with simple ways to administer medications safely (i.e. add 4mg of norepinephrine to a 1L fluid bag to create a concentration of 4mcg/mL which may be used as an infusion).
- Unreliable monitoring requires careful attention to physical examination and verification of clinical signs.
- Correction of a trainee in the setting of an error requires direct correction in a respectful manner, particularly when cultural barriers exist.
- Resources such as ventilators for critical illness may be scarce in these environments, and thoughtful planning must be made ahead of time for how to manage these challenging situations.

**Simulation 3: Pre-eclampsia in a low-resource setting**

The following teaching points may be used for debriefing following this scenario:

- Pre-eclampsia may be recognized by hypertension, proteinuria, thrombocytopenia, cerebral disturbance, impaired liver function, and other clinical signs. Aggressive treatment with antihypertensives should start early.
- Effective communication with patient, team members and consultants is critical but may be impaired with a language barrier.
- Occasionally trainees working in low-resource settings may be asked to practice outside of the comfort level or scope, which presents significant ethical considerations.
- Decision-making may be more challenging with resource-limitations, such as lack of blood availability, cost/effort required to give blood, lack of staff to manage resuscitation of mother and neonate simultaneously.
