# Supplementary material for: The Preparing Residents for International Medical Experiences (PRIME) Simulation Workshop: Equipping Surgery and Anesthesia Trainees for International Rotations
Source: MedEdPORTAL. 2021 Feb 11;17:11088. doi: 10.15766/mep_2374-8265.11088 (PMC7880254; doi:10.15766/mep_2374-8265.11088)
Supplement: Supplementary file 1 — Simulation 1.docxSimulation 2.docxSimulation 3.docxSimulation 2 Lab Values.docxSimulation 3 Lab Values.docxResident Self-Assessment.docxCritical Actions Checklist.docxDebriefing Guide.docxSimulation Evaluation.docx [file mep_2374-8265.11088-s001.zip › I. Simulation Evaluation.docx]

Appendix I. Post-rotation simulation evaluation.

| **Please indicate your agreement or disagreement with the following statements** | Responses |
| --- | --- |
| **Scenario 1** – Mass casualty requiring triage of patients and resources with loss of power during resuscitation:  "This scenario was realistic for the clinical environment I observed or experienced at Kijabe Hospital." | Strongly agree Agree Neither agree nor disagree  Disagree Strongly disagree |
| Scenario 1-- "This scenario prepared me for clinical experiences at Kijabe Hospital." | Strongly agree Agree Neither agree nor disagree  Disagree Strongly disagree |
| **Scenario 2** – Swahili-speaking parturient with eclampsia requiring C-section and induction with drugs common to a low-resource setting.  "This scenario was realistic for the clinical environment I observed or experienced at Kijabe Hospital." | Strongly agree Agree Neither agree nor disagree  Disagree Strongly disagree |
| "This scenario prepared me for clinical experiences at Kijabe Hospital." | Strongly agree Agree Neither agree nor disagree  Disagree Strongly disagree |
| **Scenario 3** – Exploratory laparotomy for abdominal sepsis with ICU unavailable with anesthesiologist supervising a student anesthetist who performs esophageal intubation.  "This scenario was realistic for the clinical environment I observed or experienced at Kijabe Hospital." | Strongly agree Agree Neither agree nor disagree  Disagree Strongly disagree |
| "This scenario prepared me for clinical experiences at Kijabe Hospital." | Strongly agree Agree Neither agree nor disagree  Disagree Strongly disagree |
